# Synergy of therapeutic vaccination against HPV16 oncogenic proteins and standard chemotherapeutics

**DOI:** 10.1186/2051-1426-2-S3-P56

**Published:** 2014-11-06

**Authors:** Cornelis J Melief, Marij JP Welters, Tetje van der Sluis, Helene van Meir, Suzanne van Duikeren, Suzanna Huppelschoten, Ekaterina S Jordanova, Judith Kroep, Gemma Kenter, Vincent Smit, Ferry Ossendorp, Bob van de Water, Mariette van Poelgeest, Jacobus Burggraaf, Ramon Arens, Sjoerd H van der Burg

**Affiliations:** 1 Leiden University Medical Center, Leiden, Netherlands; 2 Leiden Amsterdam Center for Drug Research, Netherlands; 3 Obstetrics & Gynecology, VU University Medical Center, Amsterdam, The Netherlands, Amsterdam, Netherlands; 4 Center for Gynecologic Oncology Amsterdam, Netherlands; 5 Center for Human Drug Research, Netherlands

## 

We previously developed a synthetic long peptide (SLP) vaccine against HPV16 oncoproteins that induced lesion regression in patients with HPV16+ high-grade vulvar intraepithelial neoplasia, correlated with strong vaccine-prompted HPV16-specific T cell responses. In patients with HPV16-induced metastatic cervical cancer, vaccine-induced T cell responses were weaker and did not result in improved clinical outcome. In a preclinical HPV16 E6/E7+ mouse tumor model we studied the efficacy of SLP vaccination combined with chemotherapy. Mice that received either peptide vaccination or chemotherapy showed only temporary tumor regression. Importantly, combined chemo-immunotherapy induced complete tumor eradication in 75% of the mice, which was associated with a strong tumor influx of vaccine specific tumor necrosis factor alpha (TNFα) and interferon gamma (IFN-γ) producing CD8+ CTLs. Tumor cells incubated with TNFα and IFN-γ, together with cisplatin, enhanced their chemokine expression and SLP vaccine-induced CTLs appeared to migrate earlier into the tumor beds. Combination treatment *in vitro *caused a decrease in proliferation of tumor cells and TNFα-induced enhancement of cisplatin- mediated tumor cell death, accompanied by increased expression of pro-apoptotic molecules. SLP vaccination together with carboplatin and paclitaxel, a standard combined chemotherapy, caused marked decline in the abnormally high numbers of myeloid cells in blood and tumor in the mouse model, again associated with synergy in tumor eradication. Hence, standard chemotherapy promotes the effects of SLP vaccination by better attraction of T cells into tumors, greater sensitivity of tumors to TNFα-mediated apoptosis, and better expansion of T cells through depletion of myeloid derived suppressor cells without suppression of T cells, allowing synergy in tumor eradication. A clinical pilot study on the composition of blood leukocytes in late stage cervical cancer patients also revealed high numbers of myeloid cells, associated with low T cell responses, indicating an immunosuppressed status. When these patients were treated with carboplatin-paclitaxel chemotherapy their immune profile was normalized to that of healthy subjects. Therefore a clinical trial was performed in which late-stage cervical cancer patients were treated with standard chemotherapy in combination with HPV16 SLP vaccination. Immunomonitoring confirmed the beneficial effect of myeloid cell depletion associated with a robust induction of HPV16-specific T cell responses that were sustained throughout several cycles of chemotherapy.

